# Optimizing cardioneuroablation candidate selection: a case report using tilt-table testing with Russo’s Fast Italian Protocol

**DOI:** 10.1093/ehjcr/ytaf330

**Published:** 2025-07-18

**Authors:** Dorina Stangl, Veronica Buia, Janusch Walaschek, Dirk Bastian, Laura Vitali-Serdoz

**Affiliations:** Medizinische Klinik I, Klinikum Fürth, Academic Teaching Hospital of the Friedrich-Alexander-University Erlangen-Nürnberg, Jakob-Henle Str. 1, Fürth 90766, Germany; Medizinische Klinik I, Klinikum Fürth, Academic Teaching Hospital of the Friedrich-Alexander-University Erlangen-Nürnberg, Jakob-Henle Str. 1, Fürth 90766, Germany; Medizinische Klinik I, Klinikum Fürth, Academic Teaching Hospital of the Friedrich-Alexander-University Erlangen-Nürnberg, Jakob-Henle Str. 1, Fürth 90766, Germany; Medizinische Klinik I, Klinikum Fürth, Academic Teaching Hospital of the Friedrich-Alexander-University Erlangen-Nürnberg, Jakob-Henle Str. 1, Fürth 90766, Germany; Medizinische Klinik I, Klinikum Fürth, Academic Teaching Hospital of the Friedrich-Alexander-University Erlangen-Nürnberg, Jakob-Henle Str. 1, Fürth 90766, Germany

**Keywords:** Case report, Head-up tilt-table test (HUTT), Fast Italian Protocol (FIP), Cardioneuroablation

## Abstract

**Background:**

Cardioneuroablation (CNA) has emerged as a promising therapeutic alternative to conventional pacing therapy for vasovagal syncope (VVS). Accurate patient selection and diagnosis are crucial for achieving optimal outcomes. The lack of a universally accepted protocol specifically tailored for selecting patients for CNA presents a gap in the current guidelines.

**Case summary:**

This case is about a 44-year-old male with recurrent severe syncope. Head-up tilt-table testing utilizing the newly established Fast Italian Protocol (FIP) by Russo *et al.* revealed a cardioinhibitory vasovagal response, traditionally managed with pacemaker therapy. The patient, however, opted for CNA. Targeted radiofrequency ablation of the ganglionated plexi in the right and left atria was successfully performed. Clinical follow-up at 12 months post-ablation revealed no recurrence of syncopal episodes.

**Discussion:**

This case highlights the utility of tilt testing and the efficacy of the FIP as an efficient diagnostic tool for evaluating cardioinhibitory vagal responses. It supports its role in patient selection for CNA and additionally presents a promising option for a non-invasive follow-up assessment, though further investigation is needed. Its simplicity and efficiency position it as a promising candidate for standardization as the primary diagnostic tool in CNA workflows.

Learning points

- *Effectiveness of the Fast Italian Protocol (FIP)*: FIP offers a time-efficient alternative to traditional tilt testing protocols while maintaining diagnostic accuracy for vasovagal syncope.
- *Guiding candidate selection for cardioneuroablation (CNA)*: It aids in identifying suitable candidates for cardioneuroablation, potentially reducing unnecessary pacemaker implantation.
- *Importance of pre-procedure evaluation*: FIP may serve as a useful tool for pre-procedural screening in CNA patients.

## Introduction

Vasovagal syncope (VVS) is a common cause of recurrent fainting, impacting quality of life and sometimes requiring pacemaker therapy in refractory cases, particularly with a strong cardioinhibitory component. While younger individuals are more prone to cardioinhibitory VVS, the mixed form becomes prevalent up to age 50.^1^ To avoid pacemaker implantation in this group, cardioneuroablation (CNA) has emerged as an alternative, targeting vagal ganglionated plexi to modulate parasympathetic activity. Despite growing interest in CNA, precise candidate selection remains a challenge.^2,3^ Head-up tilt-table testing (HUTT) remains a key diagnostic tool, with the Fast Italian Protocol (FIP) offering a more time-efficient alternative. We present a 44-year-old male with recurrent severe syncope, where FIP guided both diagnosis and treatment, underscoring its potential in identifying CNA candidates.

## Summary figure

**Figure:**
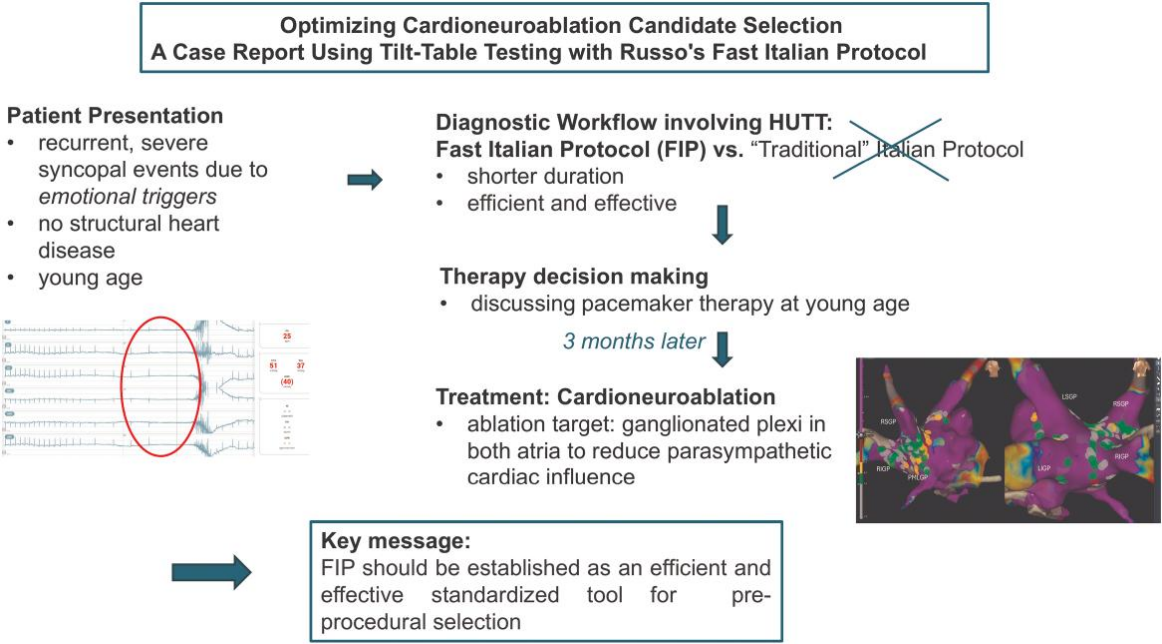


## Case presentation

A 44-year-old male came to our attention while visiting his 75-year-old mother, who was undergoing intensive care unit (ICU) monitoring following the implantation of a permanent pacemaker due to haemodynamically unstable asystole. During his visit, the patient experienced dizziness and loss of consciousness. Given the patient’s location in the ICU, immediate medical intervention was initiated, including continuous monitoring and brief cardiopulmonary resuscitation, which was not required for a prolonged period as the patient spontaneously regained consciousness and orientation.

**Figure 1:**
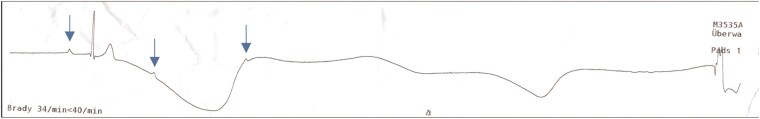
Monitoring during the loss of consciousness revealed sinus bradycardia, intermittent third-degree atrioventricular block, followed by asystole due to sinus arrest. Cardiopulmonary resuscitation was initiated but not prolonged, as circulation quickly returned spontaneously.

The monitoring revealed initial sinus bradycardia, followed by third-degree atrioventricular (AV) block and subsequently asystole due to sinus arrest (*Figure 1*).

A detailed assessment of the patient’s medical history revealed no prior use of medication and a history of multiple syncopal episodes occurring over several years, consistently triggered by emotional stress. The initial event occurred at the age of 38 during a period of pronounced work-related stress and was preceded by prodromal symptoms including lightheadedness and dizziness.

**Figure 2:**
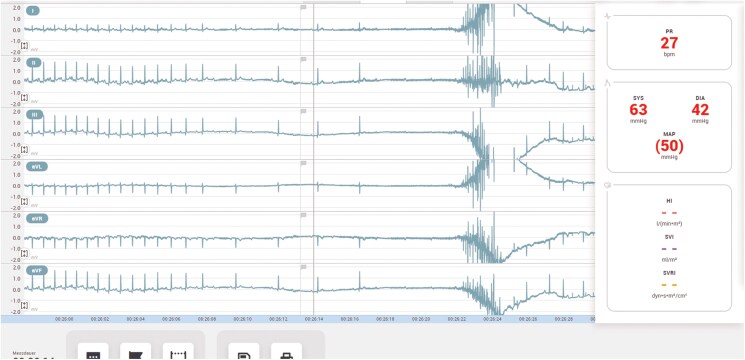
Functional atrioventricular block observed during the head-up tilt-table testing, characterized by asystole due to third-degree atrioventricular block, preceded by a slowing of the P wave rate. This was accompanied by hypotension and a syncopal event.

**Figure 3:**
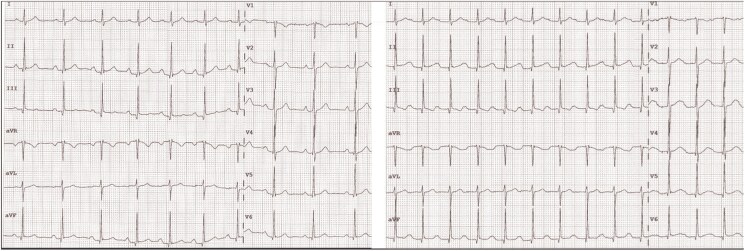
The electrocardiogram on the left side displays the rhythm before the atropine test, showing sinus rhythm at 77 b.p.m. The electrocardiogram on the right side, recorded after atropine administration, demonstrates an increase in heart rate to 105 b.p.m.

After prolonged electrocardiogram (ECG) monitoring in the ICU, where the patient remained haemodynamically stable, he was transferred to the cardiology ward. Reversible causes, including electrolyte imbalances and Lyme carditis (*Borrelia burgdorferi* antibodies were negative), were ruled out. The physical examination revealed no pathological findings. Transthoracic echocardiography, coronary angiography, and cardiac magnetic resonance imaging showed no structural abnormalities. Given suspected VVS, a HUTT was performed using the modified FIP, which shortens both the passive and active phases while maintaining diagnostic accuracy.^4^ In this approach, the passive phase of the tilt-table test is shortened from 15–10 min compared to the traditional protocol. Additionally, the active phase following nitroglycerin administration (0.3 mg sublingually) is reduced from 15 to 10 min. Similar to Russo *et al*., the Task Force monitor (CNSystem, Graz, Austria) was used to perform the HUTT. During tilt testing, the patient once again experienced syncope preceded by dizziness, closely resembling the initial clinical episode. As documented, the syncope was accompanied by sinus bradycardia followed by asystole due to third-degree AV block, confirming a cardioinhibitory response (*Figure 2*). An atropine test (0.04 mg/kg) showed an adequate heart rate increase, indicating intact autonomic activity^5^ and suggesting syncope was likely due to parasympathetic overactivation (*Figure 3*).

The patient was informed of the European Society of Cardiology guidelines recommending pacemaker implantation for individuals over 40 years old with severe VVS but declined this option. Following a patient-centred decision-making process, CNA was offered as an alternative treatment and was performed three months after the initial hospital stay.^6^

Cardioneuroablation involves ablating ganglionated plexi in both atria to reduce parasympathetic cardiac influence, which causes AV block, sinus bradycardia, or sinus arrest.

Pre-ablation measurements showed first-degree AV block and supra-Hisian AV delay.

**Figure 4:**
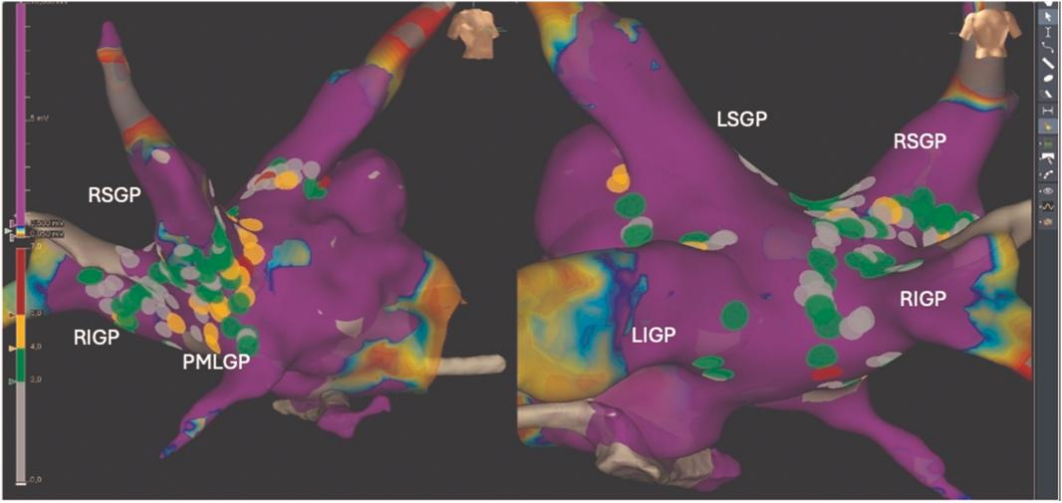
Electroanatomic map of the left atrium post-radiofrequency ablation targeting the ganglionated plexi (right superior ganglionated plexi, right inferior ganglionated plexi, left superior ganglionated plexi, left inferior ganglionated plexi, posterior medial left ganglionated plexus). Lesion size index is indicated by colour.

3D electroanatomic mapping was conducted using a high-density multi-electrode mapping catheter (HD Grid™, Abbott, Chicago, IL, USA) with the EnSite Precision™ system (Abbott). To detect the locations of ganglionated plexi, an anatomical approach was combined with an examination of electrogram morphology. The exploration of electrogram morphology entails identifying multicomponent signals at the site of ganglionated plexi. These fractionated endocardial signals indicate the entry of neural fibres into myocardial tissue, leading to inhomogeneous conduction.^7^ A fractionation mapping tool was utilized to identify these sites with more than four deflections, especially those with higher amplitudes (>0.7 mV).^8^ Key targets included the left and right superior and inferior ganglionated plexi (LSPG and LIGP) as well as the right superior and inferior ganglionated plexi (RSPG and RIGP) (*Figure 4*). A right-sided approach targeted the RSPG and posterior medial left ganglionated plexus (PMLGP).^9^

Oral anticoagulation was recommended for 2 months post-ablation.

**Figure 5:**
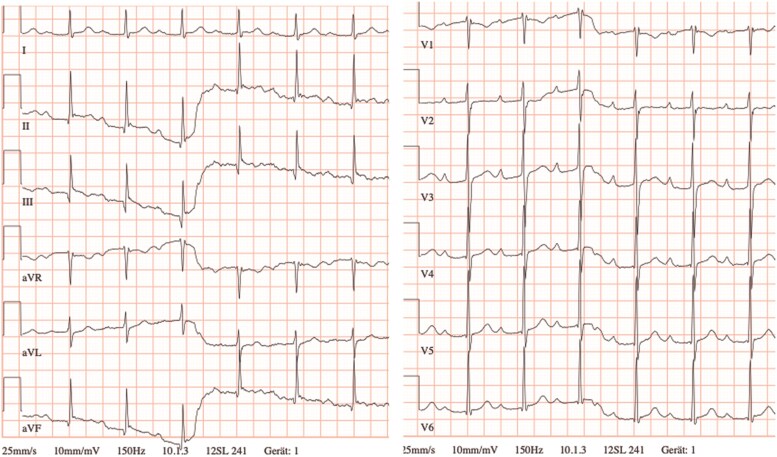
Electrocardiogram 1 day after cardioneuroablation showing sinus rhythm with an elevated resting heart rate of 88 b.p.m. and first-degree atrioventricular block with a PQ interval of 298 ms.

The ECG on the following day showed sinus rhythm with an elevated resting heart rate of up to 88 b.p.m. and a prolonged PQ interval (*Figure 5*).

The atropine test demonstrated no significant increase in heart rate, with a maximum of 99 b.p.m., and persistent PQ interval prolongation, indicative of decremental conduction over the AV node.

Follow-ups at 3 and 12 months with clinical evaluation and Holter ECG showed no asystole or AV block. The patient remained asymptomatic, with no syncope or stress-induced episodes, reporting improved quality of life—suggesting successful ganglionated plexi modulation.

## Discussion: Fast Italian Protocol for diagnosis and pre-procedural evaluation

Head-up tilt-table testing is widely used to differentiate reflex syncope subtypes and identify patients over 40 with asystole who may require pacing. With CNA gaining recognition, optimizing diagnostic tools for candidate selection is crucial. In the most recently published scientific statement about CNA, HUTT is included as part of autonomic function assessment, but no specific recommendations regarding which protocol to use were provided.^3^ While not yet widely adopted, the FIP offers a quick, standardized approach with potential for broader clinical use.

Age is crucial in selecting candidates for CNA, as cardioinhibitory patterns are more common in younger patients, while vasodepressor responses dominate in older ones. This emphasizes the need for thorough HUTT testing to identify appropriate candidates.^1^

The FIP reduces testing time while maintaining diagnostic accuracy, shortening the duration from approximately 40–25 min compared to the traditional version of the test. Russo *et al*.’s trial showed that the traditional HUTT had a 17.3% positivity rate for cardioinhibitory syncope, while the FIP increased it to 20.9%. The passive phase positivity remained low (2.0% vs. 1.8%), confirming the efficiency of the shorter test.^4^ Median time to positivity is 3–5 min, making longer testing unnecessary.^10^ Shortening both phases may prevent misclassification of cardioinhibitory syncope as delayed orthostatic hypotension.

Moreover, recent scientific statements on CNA outline follow-up methods like carotid sinus massage, HUTT, and prolonged ECG, but lack a clear post-procedural assessment strategy.^3^ Post-CNA autonomic imbalances, such as nerve sprouting or parasympathetic reinnervation, may lead to recurrence of syncopal events or a shift to vasodepressive syncope.^11^ Russo’s FIP could provide a standardized post-procedural workflow and insights into pathophysiology after CNA.

## Conclusion

In this case, the FIP proved to be an efficient and effective tool for diagnosing cardioinhibitory responses and guiding the selection of candidates for CNA.

Further research is needed to establish FIP as a standardized tool for pre-procedural patient selection. In addition, future studies should evaluate its potential role in post-procedural follow-up and long-term monitoring.

## Data Availability

The data underlying this article will be shared on reasonable request to the corresponding author.

## Lead author biography

Dr Dorina Stangl is a cardiology fellow specializing in electrophysiology at Klinikum Fürth, Bavaria, Germany. The electrophysiology department focuses on vasovagal syncope and innovative diagnostic protocols, such as the Fast Italian Protocol. Dr Stangl has contributed to establishing a structured diagnostic workflow for syncope evaluation. Her particular research interests include optimizing patient selection for cardioneuroablation (CNA) and advancing non-invasive diagnostic methodologies.

## Notes

**Consent:** The patient provided written informed consent for the publication of the case details and associated images. This case report adheres to the ethical standards outlined by the Committee on Publication Ethics (COPE).

**Funding:** This work was not supported by others—no funding.
